# Effectiveness, barriers, and facilitating factors of strategies for active delabeling of patients with penicillin allergy labels: a systematic review

**DOI:** 10.1007/s15010-025-02689-4

**Published:** 2025-11-20

**Authors:** Hannah Nürnberg, Claudia Maria Denkinger, Tabea Krause, Lars Oetken, Sophie Rauer, Amelie Rapp, Lisa Marie Kern, Torsten Hoppe-Tichy, Tilman Schöning, Elham Khatamzas, Benedict Morath

**Affiliations:** 1 Hospital Pharmacy, University Hospital Heidelberg, Im Neuenheimer Feld 670, 69120 Heidelberg, Germany; 2 Department of Infectious Diseases and Tropical Medicine, University Hospital Heidelberg, Heidelberg, Germany; 3 German Centre of Infection Research, Partner site, Heidelberg, Germany

**Keywords:** Penicillin allergy, Delabeling, Implementation, Antibiotic stewardship

## Abstract

**Background:**

Penicillin allergy is the most frequently reported allergy in hospitalized patients, although rarely confirmed. Given the negative outcomes associated with incorrect penicillin allergy diagnoses, implementation of programmatic delabeling strategies is important for successful antibiotic stewardship. This systematic review evaluates the effectiveness of different delabeling strategies and highlights elements and settings that facilitate or constrict their implementation.

**Methods:**

Following the PRISMA-statement PubMed/MEDLINE, EMBASE, Cochrane Library, and grey literature databases “Worldcat” and “OpenGrey” were searched for studies reporting on interventions to identify, evaluate, or rule out incorrect penicillin allergy in hospitalized adults. Data extraction included settings, intervention types, and their effectiveness, barriers, facilitators, and regulatory factors.

**Results:**

This review included 42 studies, involving 6269 patients undergoing penicillin allergy delabeling, with 5017 (80%) successfully delabeled. Effectiveness varied from 32% to 99%. Skin testing had the highest success rate at 87%, followed by a combination of skin testing and oral challenge (84%), and direct oral challenges (77%). Direct delabeling alone had a lower effectiveness (67%), which improved to 80% when paired with oral challenges. Key facilitators included standardized algorithms and involvement of interdisciplinary teams. Barriers included patient refusal, staff resistance, time constraints, and financial limitations, particularly in smaller hospitals.

**Conclusion:**

Dependent on financial and staffing resources, oral challenges and direct delabeling after risk stratification may be alternatives to resource-intensive methods, such as skin testing, for successful delabeling. To address barriers to widespread delabeling implementation, patient and provider education must be improved, interprofessional collaboration enhanced, and standardized protocols developed.

**Supplementary Information:**

The online version contains supplementary material available at 10.1007/s15010-025-02689-4.

## Introduction

Penicillin allergy is the most frequently reported drug allergy in medical records with 5% to 15% of hospitalized patients claiming to be allergic, though more than 90% of those will not have a confirmed allergy [1–3]. This mislabeling can lead to a lifetime avoidance of beta-lactam antibiotics, resulting in increased utilization of less effective second-line antibiotics and an increased risk of treatment failure and mortality [4–6]. Alternative antibiotics are associated with more adverse drug reactions, longer hospital stays, higher healthcare costs, and increased rates of antibiotic resistance [7–11]. Large studies show that patients with documented penicillin allergies have higher rates of *Clostridioides difficile*, *methicillin-resistant staphylococcus aureus* (MRSA) and *vancomycin-resistant enterococci* (VRE) infections or colonization [12, 13]. Penicillin allergies also appear to have significant psychological effects on patients [14]. Given these challenges, structured testing for penicillin allergy is essential before prescribing alternative antibiotics to ensure effective antibiotic stewardship. Implementing delabeling strategies in clinical practice requires consideration of each setting’s unique characteristics, including structural, personnel, and regulatory factors [15]. The competencies of healthcare professionals involved, such as pharmacists, nurses, physicians, and clinical specialists, may impact the feasibility of these strategies.

Previously published reviews have focused on the effectiveness and safety of delabeling strategies and their feasibility of implementation by different professional groups [16–18]. Other reviews have conducted a cost analysis [10]. This systematic review is not only intended to expand the perspective on effectiveness and feasibility, but also to summarize structures and factors that enable, facilitate or restrict delabeling strategies in different settings.

## Methods

This systematic review adheres to the Preferred Reporting Items for Systematic Reviews and Meta-Analyses (PRISMA) guideline [19]. The detailed methodology is described in the review protocol [20]. As not all studies reported the numbers of identified, eligible, or tested patients, effectiveness outcomes were extracted and calculated in relation to the included patients for the respective intervention to allow consistent comparison across studies. Facilitators and barriers influencing the implementation of penicillin allergy delabeling strategies were categorized according to the Consolidated Framework for Implementation Research (CFIR) [21].

### Eligibility criteria

Prospective studies involving adult patients (18 years and older) with a documented penicillin allergy in any hospital setting, i.e., inpatients or outpatient clinics, were included. No particular study design was excluded. Only original work published in English or German were considered.

Eligible studies needed to focus on measures to identify, evaluate, or exclude false penicillin allergy diagnoses. The primary outcome was the effectiveness of the delabeling intervention, measured by the number of patients cleared from their penicillin allergy diagnosis due to the respective delabeling intervention. Secondary outcomes had to expand the perspective on implementation and included (1) barriers and facilitators in implementing delabeling interventions and (2) measurable healthcare outcomes related to penicillin allergy, such as rates of postoperative infections, length of hospital stays, treatment costs, and use of broad-spectrum antibiotics. For inclusion, the studies only had to report on the primary outcome.

### Search strategy

The databases PubMed/MEDLINE, EMBASE and Cochrane Library were searched for peer-reviewed literature from 1992 to September 3rd 2024. To ensure completeness, the databases “Worldcat” and “OpenGrey” were searched for grey literature. The search strategies used for the different databases are provided in Table S1 in the supplementary material.

### Study selection process

All identified publications were collected using MS Excel^®^ (Microsoft, Richmond, USA) and duplicates resulting from searches on different databases were removed prior to the screening process. Titles and abstracts were reviewed by three independent reviewers for the inclusion criteria.

Full text screening was performed by two reviewers on the basis of consensus. Discrepancies were resolved by a third reviewer.

### Data extraction

Data were extracted to a predefined Excel sheet by one reviewer and reviewed by another. Any occurring discrepancies during the data collection process were resolved by consensus or by a third reviewer. The extracted data included information on the study design, specific inpatient setting, type of intervention (e.g. risk stratification, oral challenge, skin testing or combinations) as well as the professional groups involved in the intervention and their influence on feasibility. The effectiveness of the interventions was determined by the number of patients who were cleared from their penicillin allergy label or switched to a beta-lactam antibiotic. To assess transferability, regulatory factors that significantly influenced the feasibility of the intervention were extracted. All circumstances and structures that influenced the implementation of the intervention were identified as barriers and facilitators.

### Bias assessment

To assess reliability of the included studies, a bias assessment was carried out by two reviewers using the Newcastle-Ottawa scale [22]. Disagreements were resolved by a third reviewer. The scale has three categories: Selection, comparability, and outcome. The application of the scale and necessary reported content are available in the supplementary data.

### Data synthesis

A descriptive analysis was performed as this review intends to comprehensively characterize available penicillin allergy delabeling strategies. Effectiveness was assessed based on percentage of patients in the studies that were successfully delabeled as a result of the intervention. For this, effectiveness was stratified based on the chosen intervention, and in relation to the risk group. To illustrate the effectiveness of the various delabeling interventions, average effectiveness was calculated by adding up the number of delabeled patients across all studies and subsequently divided by the total number of patients included in those studies. To support interpretation of the pooled effectiveness, key study characteristics such as intervention type, clinical setting, and patient risk group were narratively summarized.

## Results

### Study inclusion

**Fig. 1:**
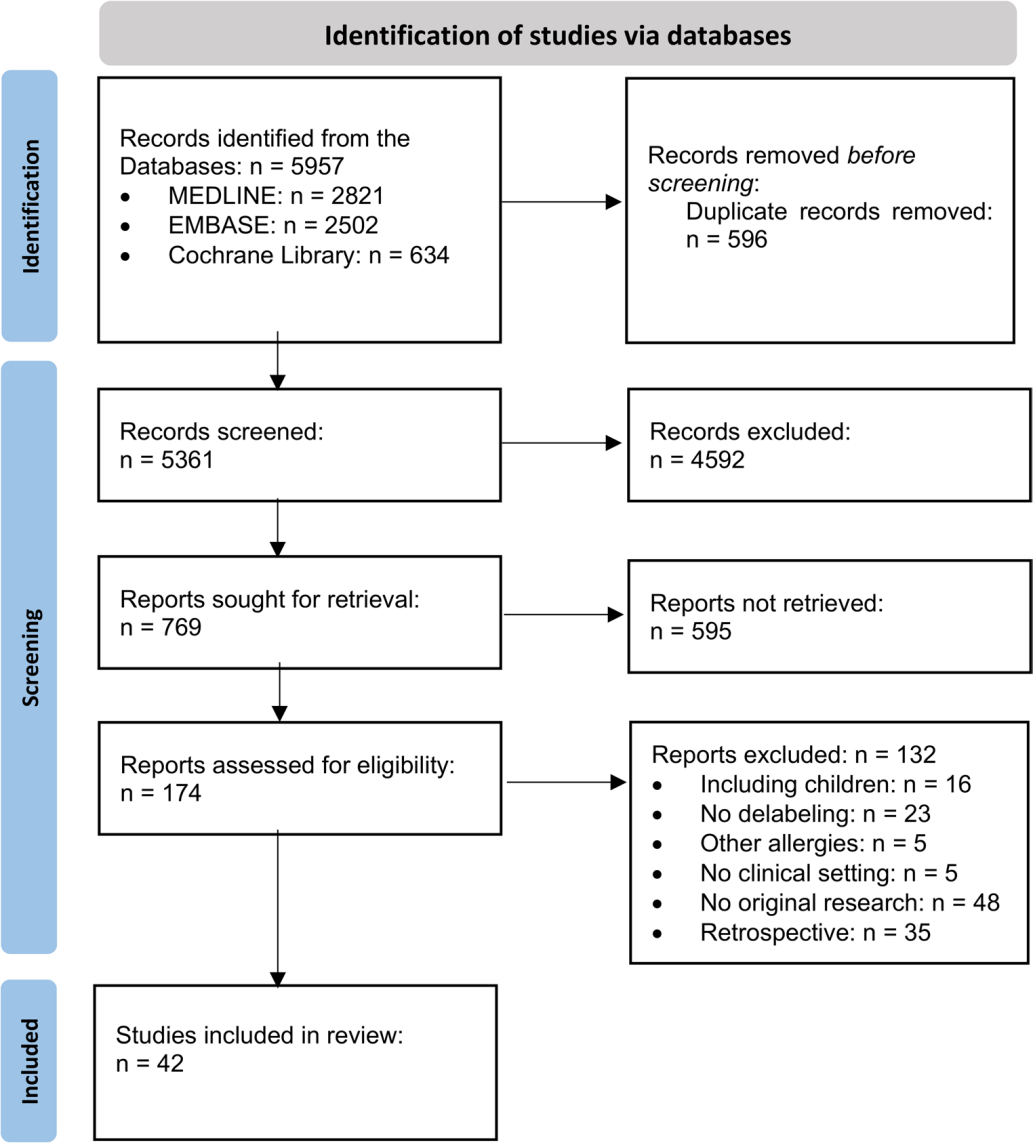
Study selection process

A total of 5957 articles were identified, with 596 duplicates removed prior to screening. The screening of 5361 See Figure 1 titles and 769 abstracts resulted in 174 full texts being assessed for inclusion. Reasons for exclusion were a retrospective study design (*n* = 35), inclusion of children and adolescents under 18 (*n* = 16), no original research (*n* = 48), no hospital setting (*n* = 5), inclusion of patients with allergies other than penicillin allergy (*n* = 5), or penicillin allergy testing as part of initial diagnoses (*n* = 23). Ultimately, 42 studies were included in this systematic review (Fig. 1).

### Characteristics of included studies

Most of the included studies were small, single centre cohort studies. The studies were conducted in eight different countries with a majority in the United States (*n* = 18), Australia (*n* = 6), and Canada (*n* = 5) (Table 1). There was only one international multicentre study that was performed in the USA, Australia, and Canada [23]. The delabeling interventions were carried out in different clinical inpatient settings, mostly surgical, oncology (*n* = 6), internal or general medicine (*n* = 9) wards (*n* = 5) or during surgical pre-assessment (*n* = 5). In 19 studies the setting was not specified, beyond the included patients being “inpatients” or “hospitalized” (Table 1).

A total of 5017 of 6269 participating patients (80%) were delabeled following penicillin allergy delabeling interventions, including skin tests followed by oral challenge (*n* = 2487), direct delabeling using patients’ history, interviews or questionnaires (*n* = 870), direct oral challenges (*n* = 1028), skin testing (*n* = 189), and outpatient testing (*n *= 58). Some studies only included patients with low-risk penicillin allergy for their interventions (*n* = 9), other studies linked the intervention to current antibiotic therapy, e.g. aztreonam, or at least prioritized patients with antibiotic therapy (*n *= 12).

**Table 1:** Characteristics of included studies

Study	Year of publication	Country	Study design (As reported by study)	Clinical setting	Intervention	Patients included in the study	Number of included patients	Outcomes
R. J. Arasaratnam et al. [24]	2024	USA	Single-centre, quality improvement study	Medical unit	Direct delabeling, direct oral challenge	Patients with penicillin allergy documented in EHR	154 Patients included	Effectiveness: Number of delabeled patients (34% (*n* = 53) of patients included) Safety: Number of adverse events
J. Bodega-Azuara et al. [25]	2024	Spain	Single-centre, prospective study	Inpatient (unspecified)	Skin Test followed by oral challenge	Patients with penicillin allergy, treated with non-beta lactam antibiotic therapy	91 Patients included	Effectiveness: Number of delabeled patients (84% (*n* = 76) of patients included) Other: Number of patients receiving beta-lactams
K. Drummond et al. [26]	2024	Australia	Multicentre, prospective cohort study	Inpatient (unspecified)	Direct delabeling, direct oral challenge	Patients with type A adverse drug reaction to penicillin	488 included patients	Effectiveness: Number of delabeled patients (83% (*n* = 404) of patients included) Other: Inpatient antibiotic use post-allergy assessment
A. M. Hitchcock et al. [27]	2024	USA	Single-centre, quasi-experimental study	Orthopaedic unit (surgical)	Direct delabeling	Patients with documented beta-lactam allergy	135 patients included in pre-intervention cohort 66 patients included in post-intervention cohort	Effectiveness: Number of patients delabeled or with updated allergy (32% (*n* = 21) of patients included in post-intervention cohort) Other: Number of patients receiving cefazolin, interview length, 90-day clinical outcome
M. T. Krishna et al. [28]	2024	United Kingdom	Multicentre, prospective observational study	Medical unit, infectious diseases ward, pre-operative assessment, oncology units	Direct oral challenge	Patients with alleged or suspected reaction to any penicillin	270 patients included	Effectiveness: Number of delabeled patients (45% (*n* = 122) of patients included) Safety: Number of adverse events Feasibility: Conversion rate from screening to consent
D. Lanoue et al. [29]	2024	Canada	Single-centre, prospective intervention study	Medical unit, surgical unit	Direct delabeling, direct oral challenge	Patients with penicillin allergy	55 Patients included	Effectiveness: Number of delabeled patients (56% (*n* = 31) of patients included) Other: Resource utilization, costs of intervention
L. E. Merz et al. [30]	2024	USA	Single-centre, quality improvement study	Oncology	Outpatient testing	Patients with penicillin allergy documented in the EMR	89 Patients included	Effectiveness: Number of delabeled patients (44% (*n* = 39) of patients included) Number of patients receiving a beta-lactam Safety: Number of adverse events
G. J. Molina-Molina [31]	2024	Spain	Multicentre, prospective study	Inpatient (unspecified)	Skin Test followed by oral challenge	Patients with beta-lactam allergy documented in the EMR	249 Patients included	Effectiveness: Number of delabeled patients (75% (*n* = 186) of patients included) Other: Use of antibiotic alternatives, demographic and clinical characteristics of patients with beta-lactam allergy labels
M. T. Rose et al. [32]	2024	Australia	Multicentre, parallel, 2-arm, open-label, randomised clinical trial	Intensive care unit	Direct oral challenge	Patients with low-risk penicillin allergy (PEN-FAST Score < 3)	80 Patients included	Effectiveness: Number of patients delabeled (49% (*n* = 39) of patients included) Safety: Number of patients with adverse events Feasibility: Number of patients that consented to participate in the study
M. Sobrino-Garcia et al. [33]	2024	Spain	Single-centre, prospective study	Inpatient (unspecified)	Skin Test followed by oral challenge	Patients with beta-lactam allergy	177 patients included	Effectiveness: Number of delabeled patients (60% (*n* = 107) of patients included) Other: Reduction of alternative antibiotics, treatment costs
J. C. Y. Wong et al. [34]	2024	China	Multicentre, prospective, pragmatic study	Inpatient (unspecified)	Skin Test followed by oral challenge	Patients with low-risk penicillin allergy who completed penicillin allergy evaluation	228 patients included 75 patients included in allergist cohort 153 patients included in non-allergist cohort	Other: Difference in effectiveness (delabeling rate) and safety between allergist and non-allergists, improvement in HR-QoL of penicillin allergy evaluation (93% (*n* = 70) of patients included in allergist cohort; 94% (*n* = 144) of patients in non-allergist cohort)
M. B. Alnaes et al. [35]	2023	Norway	Single-centre, prospective study	Infectious diseases ward, allergy clinic	Direct oral challenge, Outpatient testing	Patients with self-reported or EHR-documented penicillin allergy	149 patients included	Effectiveness: Number of delabeled patients (87% (*n* = 130) of patients included)
J. Brayson et al. [36]	2023	USA	Single-centre, prospective study	Medical unit, surgical unit	Direct oral challenge	Patients with penicillin allergy and currently on or requiring antibiotic therapy	132 patients included	Effectiveness: Number of delabeled patients (100% (*n* = 132) of patients included) Safety: Number of adverse events Other: Medical records update by the general practitioner
A. M. Copaescu et al. [23]	2023	USA, Canada, Australia	Multicentre, parallel, 2-arm, noninferiority, open-label, international randomized clinical trial	Allergy clinic	Direct oral challenge, Skin Test followed by oral challenge	Patients with penicillin allergy and PEN-FAST Score < 3	378 patients included 190 patients included in intervention group 187 patients included in control group	Effectiveness: Number of delabeled patients (98% (*n* = 186) of patients included in intervention group, 99% (*n* = 186) of patients included in control group). Safety: Number of adverse events of patients.
T. S. Li et al. [37]	2023	China	Single-centre, prospective study	Inpatient (unspecified)	Skin Test followed by oral challenge	Patients with suspected penicillin allergy who underwent penicillin allergy evaluation	372 patients included	Effectiveness: Number of delabeled patients (90% (*n* = 335) of patients included) Safety: Number of adverse events Other: Use of second-line antibiotics
S. Wade et al. [38]	2023	United Kingdom	Single-centre, prospective quality improvement study	Pre-surgical assessment	Direct delabeling	Patients with reported penicillin allergy	21 patients included	Effectiveness: Number of delabeled patients (100% (*n* = 21) of patients included) Other: Antibiotic prophylaxis, update of medical records, communication to the general practitioner
M. T. DesBiens et al. [39]	2022	USA	Single-centre, prospective observational study	Inpatient (unspecified)	Direct oral challenge	Patients with penicillin allergy and antibiotic therapy	186 patients included	Effectiveness: Tolerance of the beta lactam challenge, with no adverse event (88% (*n* = 163) of patients included) Safety: Number of adverse events Other: Improvement of antibiotic use
H. Bediako et al. [40]	2022	USA	Single-centre, prospective study	Medical unit, surgical unit	Direct delabeling	Patients with documented penicillin allergy	33 patients included	Effectiveness: Number of delabeled patients (45% (*n* = 15) of patients included)
S. Livirya et al. [41]	2022	New Zealand	Single-centre, prospective cohort study	Medical unit	Direct delabeling, Direct oral challenge	Patients with penicillin allergy	150 patients included	Effectiveness: Number of delabeled patients (75% (*n* = 112) of patients included) Other: Number of relabeled patients after 6 months
K. Y. L. Chua et al. [3]	2021	Australia	Multicentre, prospective, comparative effectiveness study	Oncology	Direct delabeling, Direct oral challenge	Patients with penicillin allergy	361 patients included	Effectiveness: Number of delabeled patients (98% (*n* = 355) of patients included) Other: Antibiotic use, readmission rate, length of stay, inpatient/90-day mortality
S. Gaudreau et al. [42]	2021	Canada	Single-centre, transversal, prospective, quasi-experimental study	Inpatient (unspecified)	Skin Test followed by oral challenge	Patients with penicillin allergy receiving antibiotic therapy and who have an infection that could be treated with a penicillin	55 patients included	Effectiveness: Number of delabeled patients (47% (*n* = 26) of patients included) Other: Acceptance of pharmacist suggestions about antibiotic treatment, use of an antibiotic with a narrow spectrum of activity
Y. Ham et al. [43]	2021	USA	Single-centre, prospective study	Inpatient (unspecified)	Direct delabeling, direct oral challenge, Skin Test followed by oral challenge	Patients with penicillin allergy. Prioritization of patients with active infections whose antibiotics would change based on testing	50 patients included	Effectiveness: Number of delabeled patients (96% (*n* = 48) of patients included) Safety: Number of adverse events Other: Percentage switched to beta-lactam therapy
S. Kwiatkowski et al. [44]	2021	USA	Single-centre, prospective, quasi-experimental study	Pre-operative assessment	Direct delabeling, outpatient testing	Patients with beta-lactam allergy and a surgery where a beta-lactam antibiotic was considered first for SSI prophylaxis	87 patients included 50 patients included in no-intervention group 37 patients included in intervention group	Effectiveness: Number of allergy labels updated (100% (*n* = 37) of patients included in the intervention group) Other: 30-day surgical site infection and CDI, acute kidney injury, allergic reactions, length of stay
Y. C. Song et al. [45]	2021	USA	Single-centre, prospective pilot study	Medical unit, surgical unit, pregnancy units	Direct delabeling	Patients with penicillin allergy documented in EHR	12 patients included	Effectiveness: Number of delabeled patients (100% (*n* = 12) of patients included) Feasibility: Time spent by pharmacist
l. Steenvoorden et al. [46]	2021	Norway	Single-centre, prospective interventional study	Medical unit	Direct oral challenge	Patients with reported low-risk penicillin allergy	57 patients included	Effectiveness: Number of delabeled patients (98% (*n* = 56) of patients included) Other: Prevalence of penicillin, practicability of method
N. P. Torney et al. [47]	2021	USA	Single-centre observational, prospective cohort study	Inpatient (unspecified)	Skin Test followed by oral challenge	Patients with self-reported or documented history of a type 1 or unknown type of allergy reaction to penicillin that occurred more than five years prior to current admission	90 patients included	Effectiveness: Percentage of patients who received PAST and were transitioned to a preferred β-lactam (84% (*n* = 76) of patients included)
S. Harmon et al. [48]	2020	USA	Single-centre, prospective study	Inpatient (unspecified)	Skin Test followed by oral challenge	Patients with penicillin allergy. Prioritization of patients receiving a nonoptimal antibiotic regimen	47 patients identified 31 patients included	Effectiveness: Number of delabeled patients (87% (*n* = 27) of patients included) Other: Number of patients whose antibiotic therapy was deescalated/ provider acceptance of recommendation, cost savings
K. L. Mann et al. [49]	2020	USA	Single-centre, prospective study	Medical unit	Direct delabeling	Patients with penicillin allergy documented in the EHR. Prioritization of patients with antibiotic therapy	175 patients included	Effectiveness: Number of patients with any change to their allergy profile (76% (*n* = 133) of patients included) Other: Types of allergy changes, time from admission to interview, number of eligible patients successfully transitioned to non-carbapenem β-lactam
A. Ramsey et al. [50]	2020	USA	Single-centre, prospective study	Inpatient (unspecified)	Direct oral challenge, Skin Test followed by oral challenge	Patients with reported penicillin allergy and receiving antibiotic therapy	100 patients included 52 patients included in PAST-group 48 patients included in DOC-group	Effectiveness: Number of negative test results (91% (*n* = 91) of patients included; 85% (*n* = 44) of patients included in PST-group; 98% (*n* = 47) of patients included in DOC-group) Safety: Number of positive test results Other: Antibiotic use, cost savings, costs of skin prick test and direct challenge
J. A. Trubiano et al. [51]	2020	Australia	Multicentre, prospective cohort study	Inpatient (unspecified, oncology, outpatient cohort	Skin Test followed by oral challenge	Patients with reported penicillin allergy	622 patients included	Effectiveness: Number of negative test results (91% (*n* = 564) of patients included) Number of any positive result of a penicillin allergy test (9% (*n* = 58) of patients included); Other: Validation of risk stratification tool
M. Devchand et al. [52]	2019	Australia	Single-centre, prospective study	Inpatient (unspecified)	Direct delabeling, oral challenge, Skin test	Patients with documented penicillin allergy and antibiotic therapy	106 patients included	Effectiveness: Number of delabeled patients (38% (*n* = 40) of patients included) Other: Antibiotic use
T. du Plessis et al. [53]	2019	New Zealand	Single-centre, prospective interventional study	Inpatient (unspecified)	Direct delabeling, direct oral challenge, Outpatient testing	Patients with reported penicillin allergy	250 patients included	Effectiveness: Number of delabeled patients (80% (*n* = 199) of patients included) Safety: Number of adverse events Other: Antibiotic use, antibiotic cost, length of hospital stays, patients’ perceptions
F. Foolad et al. [54]	2019	USA	Single-centre, prospective study	Oncology	Skin Test followed by oral challenge	Patients with type 1 reaction or unknown reaction to penicillin, receiving aztreonam	49 patients included	Effectiveness: Number of patients with a negative skin prick test or oral challenge (94% (*n* = 46) of patients included) Other: Number of patients switched to beta-lactam agent, cost savings
L. Savic et al. [55]	2019	United Kingdom	Single-centre, prospective study	Pre-surgical assessment	Direct oral challenge	Patients with low-risk reaction to penicillin, occurred > 15 years ago	74 patients included	Effectiveness: Number of delabeled patients (74% (*n* = 55) of patients included) Safety: Number of adverse events Other: Surgical prophylaxis antibiotic use
M. Taremi et al. [56]	2019	USA	Single-centre, prospective, quality improvement study	Oncology	Skin Test followed by oral challenge	Patients with history of possible type 1 reactions to penicillin	100 patients included	Effectiveness: Number of delabeled patients (95% (*n* = 95) of patients included) Other: Antibiotic use
J. R. Chen et al. [57]	2018	USA	Single-centre, prospective, quasi-experimental study	Inpatient (unspecified)	Skin Test followed by oral challenge	Patients with penicillin allergy and an active order for aztreonam	136 included 59 included for active Screening (AS)-only 77 included in AS-clinical Decision support group	Effectiveness: Number of patients tested negative (39% (*n* = 53) of patients included); Number of patients tested Safety: Number of adverse events Other: Proportion of inpatients on aztreonam receiving a skin test consult, time from admission to testing completion, whether the consultation occurred in the emergency department or an inpatient unit.
Y. Moussa et al. [58]	2018	Canada	Single-centre, prospective study	Pre-surgical assessment	Skin Test followed by oral challenge	Patients with history of an allergic reaction to penicillin	194 patients included	Effectiveness: Number of delabeled patients (94% (*n* = 183) of included patients) Other: perioperative antibiotic use
A. Ramsey et al. [59]	2018	USA	Single-centre, prospective study	Inpatient (unspecified)	Skin test	Patients with penicillin allergy and antibiotic therapy	50 patients included	Effectiveness: Number of patients with a negative skin prick test (94% (*n* = 47) of patients included) Other: Number of patients switched to a penicillin-based antibiotic
J. A. Leis et al. [60]	2017	Canada	Multicentre, prospective study	Inpatient (unspecified)	Skin Test followed by oral challenge	Patients with reported beta-lactam allergy	90 patients included	Effectiveness: Number of patients receiving and tolerating preferred β-lactam therapy after negative skin testing (92% (*n* = 83) of patients included) Other: infection related clinical outcomes
J. Marwood et al. [61]	2017	Australia	Single-centre, prospective study	Emergency department	Skin Test followed by oral challenge	Patients with self-reported history of penicillin allergy	100 patients included	Effectiveness: Perceived allergy status at the end of testing (81% (*n* = 81) of patients included) Safety: Overall and individually summarized adverse events
M. E. Arroliga et al. [62]	2003	USA	Single-centre, prospective observational Study	Intensive care units	Skin test	Patients with documented penicillin allergy	96 patients included	Effectiveness: Number of patients with negative penicillin skin test (89% (*n* = 85) of patients included) Safety: Number of adverse events after changing antimicrobial therapy to beta-lactam Other: Percentage of negative tested patients changed to a beta-lactam antimicrobial
R. J. Warrington et al. [63]	2000	Canada	Single-centre, prospective study	Inpatients (unspecified)	Skin test	Patients with history of penicillin allergy	67 patients included	Effectiveness: Number of patients with negative penicillin skin test (79% (*n* = 53) of patients included) Safety: Number of adverse events as a result of beta-lactam therapy Other: Percentage of negative tested patients changed to a beta-lactam antimicrobial
**Abbreviations**: **HR-QoL**: Health-related Quality of life | **CDI**: *Clostridioides difficile* infection | **PAST**: Penicillin allergy skin test | **EHR**: electronic health record | **EMR**: electronic medical record | **SSI**: surgical side infection | **DOC**: Direct oral challenge								

### Effectiveness of delabeling interventions

**Fig. 2:**
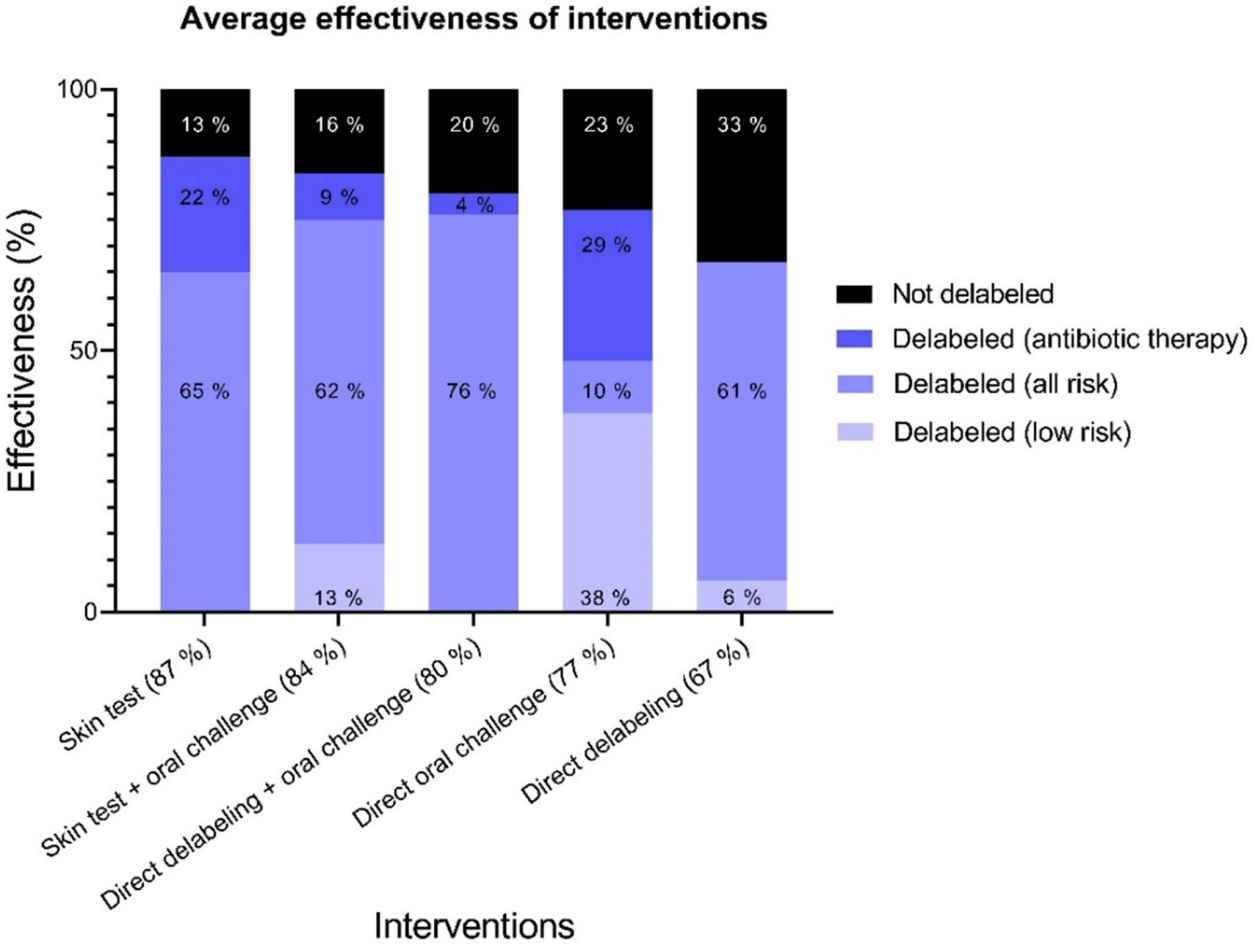
Average effectiveness of interventions and combined interventions. *Black stacked columns show proportion of not delabeled patients. Delabeled patients (blue stacked) were stratified according to different patient population of the included studies*

In total 6269 patients (*n* = 42 studies) were included for penicillin allergy delabeling interventions, with 5017 (80%) successfully delabeled. The effectiveness of the respective studies varied from 32% to 99%. The average effectiveness of the most common interventions is shown in Fig. 2. Skin testing, performed in three studies, had the highest success rate at 87% of included patients (185/213), followed by 84% (2513/2976) for a combination of skin testing and oral challenge, conducted in 17 studies. Direct oral challenges were performed in nine studies resulting in delabeling in 77% (912/1184) of the patients included for intervention. Interventions involving direct delabeling showed lower effectiveness (67% (230/344) of included patients), but effectiveness could be improved by combining it with direct oral challenges (80% (1081/1358) of included patients). The studies included different patient populations (e.g. low-risk individuals, patients on antibiotics or with infections) with varying sample sizes. Since the studies included different risk groups, Fig. 2 also shows how the average effectiveness is distributed across the various risk groups.

### Facilitating factors and barriers to penicillin allergy delabeling

Factors influencing the implementation of penicillin allergy delabeling were systematically evaluated in several areas according to the Consolidated Framework for Implementation Research (CFIR): Intervention Characteristics (e.g. complexity, adaptability), Outer Setting (e.g. external influences such as patient needs), Inner Setting (e.g. internal environment, resources, communication), Characteristics of Individuals (e.g. knowledge, beliefs, attitudes of people involved in the process), and Process (e.g. implementation actions and strategies). An overview of identified barriers and facilitators is presented in Table 2.

#### Intervention characteristics

Several studies have highlighted the relative advantage of penicillin allergy delabeling strategies such as direct delabeling and oral provocation over skin testing. Direct delabeling and oral challenge were time- and resource-efficient as they avoid the cost and complexity of skin testing [3, 30, 35, 40, 50]. Oral provocation has the advantage of excluding false positive reactions to skin tests, and reducing the duration of hospitalisation [23, 50]. The main advantage of direct delabeling was its time efficiency, which allowed integration into existing workflows, e.g. history taking by nursing staff [30, 40, 49]. However, skin testing, remained valuable in acute care settings, especially when patients were unable to provide information about their allergy history due to their clinical condition [48]. Financial issues, including reimbursement and the high cost and time required for skin testing, were particularly challenging for smaller hospitals [30, 43, 55, 60]. An important support factor identified in seven studies was the use of a simple, standardized algorithm for the intervention procedure [34, 36, 40, 41, 45, 51].

#### Outer setting

A major barrier, reported by 16 studies, was patient refusal to undergo penicillin allergy testing or to participate in studies. Reasons included feeling unwell, being overwhelmed by other diagnoses (e.g., cancer), fear of allergic reactions, anxiety about needles, or general unease [24, 38, 42, 46, 48, 53, 54, 56, 57, 59]. Recall bias, especially in elderly or frail patients, made accurate risk stratification difficult due to inaccurate memories of the initial reaction [31, 41, 44, 58]. Further, the lack of standardized guidelines and regulatory restrictions on who can perform skin tests were barriers to progress [47, 48, 53]. Additional information is provided in Table 2.

#### Inner setting

**Fig. 3:**
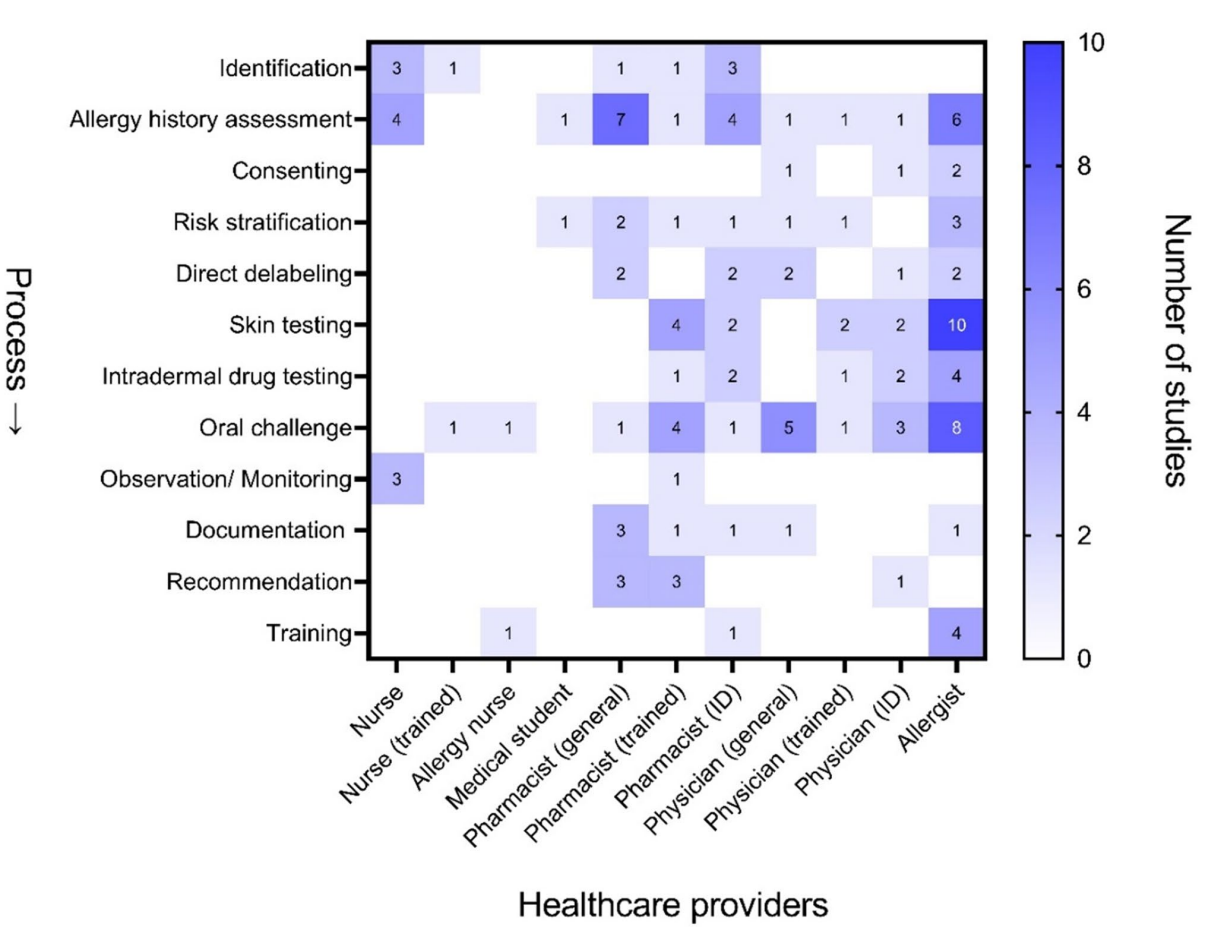
Healthcare providers involved in the delabeling process. “general”: without further specialization (prescribing pharmacist were seen as general pharmacists). “ID”: specialization in infectious diseases or antimicrobial/ antibiotic stewardship. “trained”: trained/ educated (e.g. by allergists) to perform different process steps

In terms of the inner setting, structural factors were significant (Table 2). Five studies reported that successful delabeling programs can be delivered without an allergy service, using non-specialists, pharmacists, or general physicians, most often using direct delabeling and oral provocation tests (Fig. 3) [3, 24, 29, 46, 50]. In the included studies, collaboration between key stakeholders, including specialists and non-specialists, was common [25, 36, 40, 44, 47, 54–56, 59]. While protocols often relied on allergists, collaboration between allergists and non-specialists could address this need, with allergists training and supervising the team and reducing uncertainty [28, 34, 50, 55, 58]. Hospitals with established antimicrobial stewardship programs and/or ID services might integrate delabeling into routine care more easily [3, 24, 42, 48, 52, 53, 60, 62]. However, staff shortages constrained progress and limited recruitment to weekdays [47, 54, 55]. Additionally, time constraints, short hospital stays, and early discharge were additional challenges that limited time for allergy assessments or patient education during follow-up [26, 28, 42, 49, 53, 57, 60].

#### Characteristics of individuals

Training played a key role in delabeling programs, with allergists training nurses, pharmacists, and physicians to perform tasks like interviews, skin testing, intradermal drug testing or oral challenges [24, 36, 42, 43, 47, 48, 55, 60]. Lack of confidence and knowledge in assessing, evaluating, or understanding the importance of delabeling was a common barrier among healthcare providers, further challenging delabeling efforts [34, 53]. In some cases, medical staff resisted delabeling, often because alternative antibiotics were already started before allergy evaluation, or due to concerns about the reliability of skin test, particularly for immunocompromised patients [42, 45, 54–56].

#### Process

The process could be supported by involving local champions or allergists to guide and supervise the teams [28, 34, 50, 55, 58]. Short hospital stays and early discharge often challenged planning of delabeling efforts, but some studies suggested that prioritizing patients currently receiving antibiotics could improve resource efficiency [24, 49, 57, 60]. The choice of intervention often depended on the clinical setting, with skin tests more commonly used in the ICU, where patients might be unable to provide allergy histories, while oral challenges and direct delabeling were more commonly used in surgical and internal medicine settings (see Supplementary Figure S1) [3, 24, 32, 36, 40, 41, 45, 46, 62].

**Table 2:** Facilitators and barriers for penicillin allergy delabeling mapped to CFIR domains and constructs

CFIR Domain	CFIR Construct	Facilitators	Barriers
**Intervention Characteristics**			
	*Relative Advantage*	Direct delabeling and oral challenge save time, cost, and resources [3, 30, 35, 40, 50]. Oral challenge reduces false positive reactions [23, 50]	Skin testing seen as resource-intensive and time-consuming [30, 43, 55, 60]
	*Design Quality & Packaging*	Standardized algorithms (checklists, risk stratification) [34, 36, 40, 41, 45, 51]	n.a.
	*Complexity*	Simple protocols for direct delabeling and oral provocation allow for integration into existing workflows [30, 40, 49]	Complexity of skin testing [30, 43, 55, 60]
**Outer Setting**			
	*Patient Needs & Resources*	Patient reassurance through oral challenges and skin tests [46].	Patient refusal [24, 38, 40, 42, 46, 48, 53, 54, 56, 57, 59] Lack of consent capacity and allergy information in older, critically ill or psychiatric patients [31, 32, 41, 44, 58, 62] Patient mistrust direct delabeling [24, 38, 40, 42, 46, 48, 53, 54, 56, 57, 59] Relabeling [25, 55]
	*External Policies & Incentives*	n.a.	Lack of standardized guidelines and regulatory restrictions on who can perform testing [47, 48, 53]
**Inner Setting**			
	*Structural Characteristics*	Non-specialists can perform delabeling [3, 24, 29, 46, 50]	Lack of allergy services [35, 59]. Staff shortage [47, 54, 55].
	*Networks & Communication*	Multidisciplinary collaboration [25, 36, 40, 44, 47, 54–56, 59] Allergists supervising the team to reduce uncertainty [28, 34, 50, 55, 58]	n.a.
	*Implementation Climate*	Antibiotic stewardship programs or infectious disease services [3, 24, 42, 48, 52, 53, 60, 62].	Time constraints, short hospital stay, and early discharge [26, 28, 42, 49, 53, 57, 60].
	*Readiness for Implementation*	Existing workflows (e.g. medication assessment, history taking by nursing staff) [30, 40, 49]. Clear allergy documentation, electronic prescribing system [49, 61].	n.a.
**Characteristics of Individuals**			
	*Knowledge & Beliefs*	Training of pharmacists, nurses, and physicians to perform delabeling tasks [24, 36, 42, 43, 47, 48, 55, 60].	Lack of knowledge and confidence in assessing allergies [34, 53].
	*Self-Efficacy*	Training and guidance improve staff confidence [35].	Resistance of medical staff to delabeling [42, 45, 54–56].
**Process**			
	*Planning*	Prioritization of patients most likely to benefit (e.g. already on antibiotics) for resource efficiency [24, 49, 57, 60].	n.a.
	*Engaging*	Involvement of local champions and allergists to guide the process [28, 34, 50, 55, 58]	Low patient participation (e.g. missed appointments, low survey completion) [25, 30]
	*Executing*	Tailored interventions based on clinical setting (ICU vs. surgical wards) [3, 24, 32, 36, 40, 41, 45, 46, 62].	n.a.

### Impact on healthcare

Due to the prospective study designs, most studies reported direct changes in antibiotic use following allergy testing. Patients on antibiotics were often switched to preferred beta-lactams, and other studies compared antibiotic use between delabeled and non-delabeled patients [3, 26, 37, 44, 52, 53, 62]. However, only a few studies investigated overall changes in antibiotic consumption, showing significant reductions in aztreonam use after the interventions [54, 57]. One study also reported five (10%) surgical site infections in the standard-care group, compared to none in the intervention group, of which all were associated with the use of alternative antibiotics [44].

In terms of costs, one study focused on the costs of implementing interventions [9], while others explored direct savings in antibiotic costs [3, 48, 54, 56, 57, 59]. Antibiotic costs were found to be up to 2.5 times higher in patients with confirmed allergies than in delabeled patients [33, 53].

### Bias assessment

Of the 42 included studies, no studies had a high (1–3 points), 33 studies had a moderate (4–6 points) and nine studies had a low risk (7–9 points) of bias. The results of the bias assessment are listed in Table S2 in the supplementary material. Most studies were small, single centre cohort studies and thoroughly reported on the identification of penicillin allergy labels as well as the results of the tests conducted. However, some of these studies focused on smaller patient populations (such as patients on antibiotic therapy or only low-risk patients) or often did not check whether patients had previously undergone a penicillin allergy test, which introduces a moderate risk of bias. Studies that were rated with low risk of bias were generally able to meet these criteria. Additionally, there were studies considering another factor that could influence the outcome of the delabeling process by comparing testing strategies and the implementation of the intervention by different healthcare professionals.

## Discussion

In this systematic review, we found that the effectiveness of delabeling strategies varies widely across the 42 included studies, ranging from 32% to 99%, depending on the setting and intervention. Skin testing and oral challenges were more effective (77–87%) than direct delabeling (67%). However, effectiveness varied even for similar interventions and was influenced by factors such as patient populations, sample sizes, and risk group classifications. The results suggest that the choice of intervention should be setting-specific to optimize implementation and effectiveness (see Supplementary Figure S1). Direct delabeling and oral challenges offered time-, cost- and resource-efficient alternatives to skin testing, and provided valuable options for easier integration into existing workflows. Other systematic reviews supported delabeling large patient numbers without allergy specialists or skin testing [16, 64]. Oral provocation helped to minimize false positive tests and shortened hospital stays [23, 26, 35]. In addition, direct delabeling, performed by non-specialists (e.g. pharmacists, medical students or nurses) was also effective [30, 40, 49]. However, in specific settings such as intensive care or among elderly patients, skin testing prior to penicillin administration appeared more appropriate, as these patients were often unable to provide information on their allergy history [41, 62].

Understaffing has been reported as a common challenge that may constrain the implementation of penicillin allergy delabeling interventions [24, 25, 47, 55, 60, 65]. Multidisciplinary collaboration, clear communication, and standardized workflow have been suggested to have a positive influence on implementation of penicillin allergy delabeling [25, 36, 41, 44, 45, 47, 55, 56]. In this review, pharmacists were identified to play a key role in delabeling by performing direct delabeling, oral challenges as well as skin tests after training [43, 47, 48, 57]. However, legal restrictions may limit the ability of pharmacists to perform certain procedures, especially in countries without medical prescribing rights for pharmacists [42, 66]. Many of the included studies were conducted outside Europe, and many of the pharmacists were specialized or even independent prescribers [36, 50, 53, 54, 56, 59, 60]. Given these extended competencies (e.g. prescribing authority, skin testing), the transferability to general pharmacists in countries without such tasks remains questionable. Developing safe, guideline-based interventions that can be implemented by non-specialized staff, could further empower these professions to play a key role in penicillin allergy delabeling [67, 68].

Clinicians appeared to lack the experience and skills and sometimes report to be not confident in performing delabeling [69]. Therefore, expanding delabeling training into the clinical education, and targeted education to clinical staff such as nurses, physician assistants, or medical students, could support broader implementation of delabeling and address high workload challenges [70]. As well as healthcare providers, patients themselves also play a key role in the success of delabeling strategies. Patient refusal, often due to fear of allergic reactions, or lack of motivation and understanding of the purpose of delabeling, remains a significant challenge [25, 40, 42, 46, 50, 55, 70]. Hence, simply testing and delabeling patients might be insufficient if patients do not understand or accept delabeling. Up to 36% of patients regain the penicillin label after a negative allergy evaluation [71, 72]. One included study using direct delabeling and oral challenge reported a 10% relabeling rate (4 of 41 patients) after six months, while another study found 49% of skin test-negative patients still labeled as allergic at discharge [41, 63]. Many interventions of included studies lacked follow-up data or information on whether the effect is sustained.

One follow-up study found that while 94% of patient hospital records were correctly updated after delabeling, only 37% of primary care records reflected the change [73]. Future studies should investigate in exploring ways to connect hospital and primary care to improve the sustainability of delabeling, e.g. by improving documentation and communication, and promoting patient understanding.

### Limitations

The systematic review has some limitations. Literature that is only available in other databases, published after September 3rd 2024, as well as relevant studies published in other languages than English or German may be missing. In addition, most studies are quasi-experimental, or single-centre studies, which often carry a higher risk of bias and might be limited in transferability. We have addressed this risk through a rigorous bias assessment and reported in detail on the characteristics of the respective studies to better assess transferability. All studies were conducted in developed countries and may not be applicable to middle- or low-income countries. A major limitation across the literature was the lack of standardized risk stratification for penicillin allergy, which also impacted this review. Data were heterogeneous, and comparability between studies was limited, which precluded a formal meta-analysis. The development of national or international guidelines with standardized risk stratification would facilitate the comparison of delabeling strategies. The pooled effectiveness analysis provides a general overview of delabeling success, but should be interpreted with caution. The included studies differed in terms of design, patient selection, clinical setting, and delabeling approach. These differences are described in the narrative synthesis of study characteristics and should be taken into account when interpreting the results. The pooled data provide a general indication of effectiveness, but may not fully reflect the specific contexts in which the interventions were applied.

Although most studies used quantitative methods, many still included helpful information about factors that influence implementation, such as workflows, structural conditions, or staff behavior. Even if these aspects were not the main focus, they offered useful insights into barriers and facilitators. Mapping these findings to CFIR constructs allowed for a more systematic interpretation of implementation challenges and facilitators in different settings.

## Conclusion

The effectiveness of different delabeling strategies varies, with skin testing and oral challenges demonstrating the highest success rates. A key finding from this review is the importance of using standardized algorithms and interdisciplinary approaches to support implementation of penicillin allergy delabeling. However, several barriers, including patient refusal, medical staff resistance, logistical challenges, and financial limitations constrain widespread implementation. Addressing these challenges will require both improving patient and provider education, enhancing collaboration of healthcare professionals, and developing clear protocols for delabeling. In resource-limited settings, oral challenges, and direct delabeling after standardized risk stratification may offer practical alternatives to more resource-intensive methods like skin testing. To maximize the potential of penicillin allergy delabeling and improve antimicrobial therapy in patients, interdisciplinary teams should be formed that work within clearly defined processes with standardized risk stratification.

## Supplementary Information

Below is the link to the electronic supplementary material.

**Supplementary Material 1**: **Supplementary data** 1. Definition of different delabeling strategies. 2. Bias assessment specification. **Figure S1**: Interventions across healthcare providers and settings. **Table S1**: Search strategy. **Table S2**: Detailed results of bias assessment.

**Supplementary Material 2**

## Data Availability

Data will be made available upon reasonable request.

## Declarations

### Registration and Protocol

This systematic review was not registered. The review protocol can be assessed on BMJ open [20].

## References

[1] Sacco KA, Brigham BA, Imam TJ, Burton JS. MC., Clinical outcomes following inpatient penicillin allergy testing: A systematic review and meta-analysis. 2017. 10.1111/all.13168 28370003

[2] Blumenthal KG, et al. Antibiotic Allergy Lancet. 2019;393(10167):183–98. 30558872 10.1016/S0140-6736(18)32218-9 PMC6563335

[3] Chua KYL, et al. The penicillin allergy delabeling program: A multicenter Whole-of-Hospital health services intervention and comparative effectiveness study. Clin Infect Dis. 2021;73(3):487–96. 32756983 10.1093/cid/ciaa653 PMC8326579

[4] MacLaughlin EJ, Saseen JJ, Malone DC. Costs of beta-lactam allergies: selection and costs of antibiotics for patients with a reported beta-lactam allergy. Arch Fam Med. 2000;9(8):722–6. 10927711 10.1001/archfami.9.8.722

[5] King EA, et al. Penicillin skin testing in hospitalized patients with beta-lactam allergies: effect on antibiotic selection and cost. Ann Allergy Asthma Immunol. 2016;117(1):67–71. 27211057 10.1016/j.anai.2016.04.021

[6] Stone CA Jr., et al. The challenge of de-labeling penicillin allergy. Allergy. 2020;75(2):273–88. 31049971 10.1111/all.13848 PMC6824919

[7] Shenoy ES, et al. Evaluation and management of penicillin allergy: A review. JAMA. 2019;321(2):188–99. 30644987 10.1001/jama.2018.19283

[8] Lee RU. Penicillin allergy delabeling can decrease antibiotic Resistance, reduce Costs, and optimize patient outcomes. Fed Pract. 2020;37(10):460–5. 33132684 10.12788/fp.0040 PMC7592897

[9] Powell N, Honeyford K, Sandoe J. Impact of penicillin allergy records on antibiotic costs and length of hospital stay: a single-centre observational retrospective cohort. J Hosp Infect. 2020;106(1):35–42. 32502582 10.1016/j.jhin.2020.05.042

[10] Mattingly TJ 2, et al. The cost of Self-Reported penicillin allergy: A systematic review. J Allergy Clin Immunol Pract. 2018;6(5):1649–54.e4. 10.1016/j.jaip.2017.12.033 29355644

[11] Mabilat C, et al. Improving antimicrobial stewardship with penicillin allergy testing: a review of current practices and unmet needs. JAC Antimicrob Resist. 2022;4(6):dlac116. 36415507 10.1093/jacamr/dlac116 PMC9675589

[12] Macy E, Contreras R. Health care use and serious infection prevalence associated with penicillin allergy in hospitalized patients: A cohort study. J Allergy Clin Immunol. 2014;133(3):790–6. 24188976 10.1016/j.jaci.2013.09.021

[13] Blumenthal KG, et al. Risk of meticillin resistant Staphylococcus aureus and clostridium difficile in patients with a documented penicillin allergy: population based matched cohort study. BMJ. 2018;361:k2400. 29950489 10.1136/bmj.k2400 PMC6019853

[14] Blumenthal KG, et al. Patient characteristics and concerns about drug allergy: A report from the united States drug allergy registry. J Allergy Clin Immunol Pract. 2020;8(9):2958–67. 32853819 10.1016/j.jaip.2020.08.018

[15] Skivington K et al. A new framework for developing and evaluating complex interventions: update of Medical Research Council guidance. BMJ, 2021;374:n2061. 10.1136/bmj.n2061 PMC8482308 34593508

[16] Powell N, et al. The effectiveness of interventions that support penicillin allergy assessment and delabeling of adult and pediatric patients by nonallergy specialists: a systematic review and meta-analysis. Int J Infect Dis. 2023;129:152–61. 36450321 10.1016/j.ijid.2022.11.026 PMC10017351

[17] Cooper L, et al. Safety and efficacy of de-labelling penicillin allergy in adults using direct oral challenge: a systematic review. JAC Antimicrob Resist. 2021;3(1):dlaa123. 34223072 10.1093/jacamr/dlaa123 PMC8210118

[18] Blumenthal KG, et al. Reaction risk to direct penicillin challenges: A systematic review and Meta-Analysis. JAMA Intern Med. 2024;184(11):1374–83. 39283610 10.1001/jamainternmed.2024.4606 PMC11406457

[19] Page MJ, et al. The PRISMA 2020 statement: an updated guideline for reporting systematic reviews. BMJ. 2021;372:n71. 33782057 10.1136/bmj.n71 PMC8005924

[20] Nurnberg H, et al. Effectiveness, barriers and facilitating factors of strategies for active delabelling of patients with penicillin allergy labels: a systematic review protocol. BMJ Open. 2024;14(2):e077927. 38413160 10.1136/bmjopen-2023-077927 PMC10900358

[21] Damschroder LJ et al. Fostering implementation of health services research findings into practice: a consolidated framework for advancing implementation science. Implement Sci, 2009. 4. 10.1186/1748-5908-4-50 PMC2736161 19664226

[22] Wells GA, O’Connell BSD, Peterson J, Welch V, Losos M. P Tugwell. The Newcastle-Ottawa Scale (NOS) for assessing the quality of nonrandomised studies in meta-analyses. 2013 15.06.2023]; Available from: https://www.ohri.ca/programs/clinical_epidemiology/oxford.asp

[23] Copaescu AM, et al. Efficacy of a clinical decision rule to enable direct oral challenge in patients with Low-Risk penicillin allergy: the PALACE randomized clinical trial. JAMA Intern Med. 2023;183(9):944–52. 37459086 10.1001/jamainternmed.2023.2986 PMC10352926

[24] Arasaratnam RJ, et al. Rising to the challenge: an ID Provider-Led initiative to address penicillin allergy labels at a large veterans affairs medical center. Open Forum Infect Dis. 2024;11(8):ofae396. 39130085 10.1093/ofid/ofae396 PMC11310584

[25] Bodega-Azuara J, et al. Beta-lactam allergy in patients: an antibiotic stewardship challenge. Eur J Hosp Pharm. 2024;31(4):307–13. 36564160 10.1136/ejhpharm-2022-003304 PMC11265552

[26] Drummond K, et al. Effectiveness of direct delabelling of allergy labels in type A adverse drug reactions to penicillin: a multicentre hospitalwide prospective cohort study. J Antimicrob Chemother. 2024;79(10):2640–4. 39078218 10.1093/jac/dkae270

[27] Hitchcock AM, et al. Impact of a Pharmacist-Conducted preoperative Beta-Lactam allergy assessment on perioperative Cefazolin prescribing. J Pharm Pract. 2024;37(5):1073–81. 37931904 10.1177/08971900231214581

[28] Krishna MT, et al. A multicentre observational study to investigate feasibility of a direct oral penicillin challenge in de-labelling ‘low risk’ patients with penicillin allergy by non-allergy healthcare professionals (SPACE study): implications for healthcare systems. J Infect. 2024;88(3):106116. 38331329 10.1016/j.jinf.2024.01.015 PMC10961940

[29] Lanoue D, et al. Resource utilization and cost assessment of a proactive penicillin allergy de-labeling program for low-risk inpatients. Allergy Asthma Clin Immunol. 2024;20(1):7. 38254221 10.1186/s13223-023-00864-6 PMC10804656

[30] Merz LE, et al. Development of a pipeline for removing allergy labels in patients undergoing hematopoietic stem cell transplantation. Transpl Cell Ther. 2024;30(3):e3221–32210. 10.1016/j.jtct.2023.12.012 38134971

[31] Molina-Molina GJ, et al. Delabelling beta-lactam allergy. Front Pharmacol. 2024;15:1423719. 38994200 10.3389/fphar.2024.1423719 PMC11237397

[32] Rose MT, et al. Oral challenge vs routine care to assess low-risk penicillin allergy in critically ill hospital patients (ORACLE): a pilot safety and feasibility randomised controlled trial. Intensive Care Med. 2024;50(6):913–21. 38739277 10.1007/s00134-024-07448-x PMC11164790

[33] Sobrino-Garcia M, et al. Delabeling of allergy to beta-lactam antibiotics in hospitalized patients: a prospective study evaluating cost savings. Int J Clin Pharm. 2024;46(5):1067–75. 38642250 10.1007/s11096-024-01737-7

[34] Wong JCY, et al. Prospective, Multicenter, Head-to-Head comparison between allergists versus nonallergists in Low-Risk penicillin allergy delabeling: Effectiveness, Safety, and quality of life (HK-DADI2). J Allergy Clin Immunol Pract. 2024;12(7):1801–8.e2. 38631522 10.1016/j.jaip.2024.04.010

[35] Alnaes MB, et al. A new pathway for penicillin delabeling in Norway. World Allergy Organ J. 2023;16(11):100829. 37868111 10.1016/j.waojou.2023.100829 PMC10587752

[36] Brayson J, et al. CATALYST: challenging antibiotic allergy status. J Antimicrob Chemother. 2023;78(5):1241–4. 36975000 10.1093/jac/dkad081

[37] Li TS et al. Prospective assessment of penicillin allergy (PAPA): evaluating the performance of penicillin allergy testing and post-delabelling outcomes among Hong Kong Chinese. Asian Pac J Allergy Immunol, 2023. 10.12932/AP-270922-1469 37061932

[38] Wade S, Marshall E. A pharmacist-led penicillin allergy de-labelling project within a preoperative assessment clinic: the low-hanging fruit is within reach. J Hosp Infect. 2023;139:1–5. 37343770 10.1016/j.jhin.2023.06.012

[39] DesBiens MT, Calderwood MS, Reigh EL. Expanding penicillin allergy evaluation in hospitalized patients. Am J Med. 2022;135(8):958–63.e13. 35339433 10.1016/j.amjmed.2021.12.012

[40] Bediako H, et al. Impact of an inpatient nurse-initiated penicillin allergy delabeling questionnaire. Antimicrob Steward Healthc Epidemiol. 2022;2(1):e86. 36483390 10.1017/ash.2022.55 PMC9726580

[41] Livirya S, et al. Oral amoxicillin challenge for low-risk penicillin allergic patients. Intern Med J. 2022;52(2):295–300. 32672891 10.1111/imj.14978

[42] Gaudreau S, et al. Resources assessment for penicillin allergy testing performed by pharmacists at the patient’s bedside. Ann Pharmacother. 2021;55(11):1355–62. 33703922 10.1177/10600280211002412 PMC8908455

[43] Ham Y, et al. Safety and efficacy of direct two-step penicillin challenges with an inpatient pharmacist-driven allergy evaluation. Allergy Asthma Proc. 2021;42(2):153–9. 33685561 10.2500/aap.2021.42.200128 PMC8133016

[44] Kwiatkowski S, et al. Optimizing preoperative antibiotics in patients with beta-lactam allergies: A role for pharmacy. Am J Health Syst Pharm. 2021;78(Supplement_3):S76–82. 34037708 10.1093/ajhp/zxab218 PMC8241474

[45] Song YC et al. Effectiveness and feasibility of Pharmacist-Driven penicillin allergy De-Labeling pilot program without skin testing or oral challenges. Pharm (Basel), 2021. 9(3). 10.3390/pharmacy9030127 PMC8293328 34287342

[46] Steenvoorden L, et al. De-labelling penicillin allergy in acutely hospitalized patients: a pilot study. BMC Infect Dis. 2021;21(1):1083. 34670500 10.1186/s12879-021-06794-1 PMC8527685

[47] Torney NP, Tiberg MD. Description of a pharmacist-managed/administered penicillin allergy skin testing service at a community hospital. Am J Health Syst Pharm. 2021;78(12):1066–73. 33611361 10.1093/ajhp/zxab068

[48] Harmon S, et al. The clinical and financial impact of a Pharmacist-Driven penicillin skin testing program on antimicrobial stewardship practices. Hosp Pharm. 2020;55(1):58–63. 31983768 10.1177/0018578718817917 PMC6961156

[49] Mann KL, Wu JY, Shah SS. Implementation of a Pharmacist-Driven detailed penicillin allergy interview. Ann Pharmacother. 2020;54(4):364–70. 31701755 10.1177/1060028019884874

[50] Ramsey A, et al. Direct challenges to Penicillin-Based antibiotics in the inpatient setting. J Allergy Clin Immunol Pract. 2020;8(7):2294–301. 32156611 10.1016/j.jaip.2020.02.033

[51] Trubiano JA, et al. Development and validation of a penicillin allergy clinical decision rule. JAMA Intern Med. 2020;180(5):745–52. 32176248 10.1001/jamainternmed.2020.0403 PMC7076536

[52] Devchand M, et al. Evaluation of a pharmacist-led penicillin allergy de-labelling ward round: a novel antimicrobial stewardship intervention. J Antimicrob Chemother. 2019;74(6):1725–30. 30869124 10.1093/jac/dkz082

[53] du Plessis T, et al. Implementation of a pharmacist-led penicillin allergy de-labelling service in a public hospital. J Antimicrob Chemother. 2019;74(5):1438–46. 30753497 10.1093/jac/dky575

[54] Foolad F, et al. The impact of penicillin skin testing on Aztreonam stewardship and cost savings in immunocompromised cancer patients. Open Forum Infect Dis. 2019;6(10):ofz371. 31660339 10.1093/ofid/ofz371 PMC6767966

[55] Savic L, et al. Penicillin allergy de-labelling ahead of elective surgery: feasibility and barriers. Br J Anaesth. 2019;123(1):e110–6. 30915983 10.1016/j.bja.2018.09.009

[56] Taremi M, et al. Safety, Efficacy, and clinical impact of penicillin skin testing in immunocompromised cancer patients. J Allergy Clin Immunol Pract. 2019;7(7):2185–e21911. 30928660 10.1016/j.jaip.2019.03.025

[57] Chen JR, et al. Improving Aztreonam stewardship and cost through a penicillin allergy testing clinical guideline. Open Forum Infect Dis. 2018;5(6):ofy106. 29977963 10.1093/ofid/ofy106 PMC6016425

[58] Moussa Y et al. De-labeling of beta-lactam allergy reduces intraoperative time and optimizes choice in antibiotic prophylaxis. Surgery, 2018. 10.1016/j.surg.2018.03.004 29751965

[59] Ramsey A, Staicu ML. Use of a penicillin allergy screening algorithm and penicillin skin testing for transitioning hospitalized patients to First-Line antibiotic therapy. J Allergy Clin Immunol Pract. 2018;6(4):1349–55. 29242142 10.1016/j.jaip.2017.11.012

[60] Leis JA, et al. Point-of-Care beta-Lactam allergy skin testing by antimicrobial stewardship programs: A pragmatic multicenter prospective evaluation. Clin Infect Dis. 2017;65(7):1059–65. 28575226 10.1093/cid/cix512

[61] Marwood J, et al. De-labelling self-reported penicillin allergy within the emergency department through the use of skin tests and oral drug provocation testing. Emerg Med Australas. 2017;29(5):509–15. 28378949 10.1111/1742-6723.12774

[62] Arroliga ME, et al. A prospective observational study of the effect of penicillin skin testing on antibiotic use in the intensive care unit. Infect Control Hosp Epidemiol. 2003;24(5):347–50. 12785408 10.1086/502212

[63] Warrington RJ, Lee KR, McPhillips S. The value of skin testing for penicillin allergy in an inpatient population: analysis of the subsequent patient management. Allergy Asthma Proc. 2000;21(5):297–9. 11061039 10.2500/108854100778248269

[64] Stul F, et al. Safe penicillin allergy delabeling in primary care: A systematic review and Meta-Analysis. J Allergy Clin Immunol Pract. 2024;12(9):2415–e24261. 38901618 10.1016/j.jaip.2024.06.017

[65] Narayanan PP, Jeffres MN. Feasibility, Benefits, and limitations of a penicillin allergy skin testing service. Ann Pharmacother. 2017;51(6):504–10. 28152605 10.1177/1060028017690854

[66] Cheon E, Horowitz HW. New avenues for antimicrobial stewardship: the case for penicillin skin testing by pharmacists. Clin Infect Dis. 2019;68(12):2123–4. 30281071 10.1093/cid/ciy828

[67] Tipton C, Marikar D. Guideline review: BSACI guideline for the set-up of penicillin allergy delabelling services by non-allergists working in a hospital setting. Arch Dis Child Educ Pract Ed; 2024. 10.1136/archdischild-2023-326643 39242180

[68] Wijnakker R, et al. The Dutch working party on antibiotic policy (SWAB) guideline for the approach to suspected antibiotic allergy. Clin Microbiol Infect. 2023;29(7):863–75. 37068548 10.1016/j.cmi.2023.04.008

[69] Alagoz E, et al. Barriers to penicillin allergy de-labeling in the inpatient and outpatient settings: a qualitative study. Allergy Asthma Clin Immunol. 2023;19(1):88. 37821953 10.1186/s13223-023-00842-y PMC10568923

[70] Mann J, et al. Barriers to and facilitators of delabelling of antimicrobial allergies: A qualitative Meta-synthesis. Can J Hosp Pharm. 2024;77(1):e3490. 38357298 10.4212/cjhp.3490 PMC10846797

[71] McDanel D, et al. Relabeling of penicillin drug allergy after evaluation in a drug allergy clinic. J Allergy Clin Immunol Pract. 2022;10(1):346–8. 34537400 10.1016/j.jaip.2021.09.013

[72] Gerace KS, Phillips E. Penicillin allergy label persists despite negative testing. J Allergy Clin Immunol Pract. 2015;3(5):815–6. 26143017 10.1016/j.jaip.2015.05.019

[73] Pinto T, et al. Follow-up of penicillin allergy labels 1 year after successful penicillin delabeling. Ann Allergy Asthma Immunol. 2023;130(1):80–e833. 36116749 10.1016/j.anai.2022.09.012

