# Supplementary material for: Effectiveness, barriers, and facilitating factors of strategies for active delabeling of patients with penicillin allergy labels: a systematic review
Source: Infection. 2025 Nov 20;54(2):589–605. doi: 10.1007/s15010-025-02689-4 (PMC13021699; doi:10.1007/s15010-025-02689-4)
Supplement: Supplementary file 1 — Supplementary Material 1: Supplementary data 1. Definition of different delabeling strategies. 2. Bias assessment specification. Figure S1: Interventions across healthcare providers and settings. Table S1: Search strategy. Table S2: Detailed results of bias assessment. [file 15010_2025_2689_MOESM1_ESM.pdf]

SUPPLEMENTARY

1. **Definition of different delabeling strategies:**

Delabeling strategies were defined as follows in this review. Direct delabeling refers to the removal of the penicillin allergy label based on the patient's allergy history without performing any tests. In contrast, an oral challenge involves an oral provocation test where the patient is given oral penicillin (e.g. amoxicillin). If the procedure is done without prior skin testing it is called a "direct oral challenge". Alternatively, an oral challenge can follow a skin test to confirm the results ("skin test followed by oral challenge"). Skin testing methods include skin prick test and intradermal drug testing.

2. **Bias assessment specification:**

To earn 4 points in the selection category, studies had to include a truly or partially representative population for delabeling, with non-delabeled patients from the same population, identification of penicillin allergy label had to be based on reliable records or structured interviews and effective procedures to determine penicillin allergy verification should not have been performed prior to study participation.

To earn 1 or 2 points in the comparability category, the study should report on one or more factors in study design that could influence outcome of the delabeling process (such as comparing different test methods or implementation by different healthcare professionals).

To achieve the full 3 points in the outcome category, delabeling success had to be demonstrated following a negative oral challenge or skin test, or by penicillin intake without reaction prior to study participation per documention in healthcare records, observation period of at least 30 minutes (15 minutes for skin tests) following delabeling and be conducted for at least 90 % of patients [1].

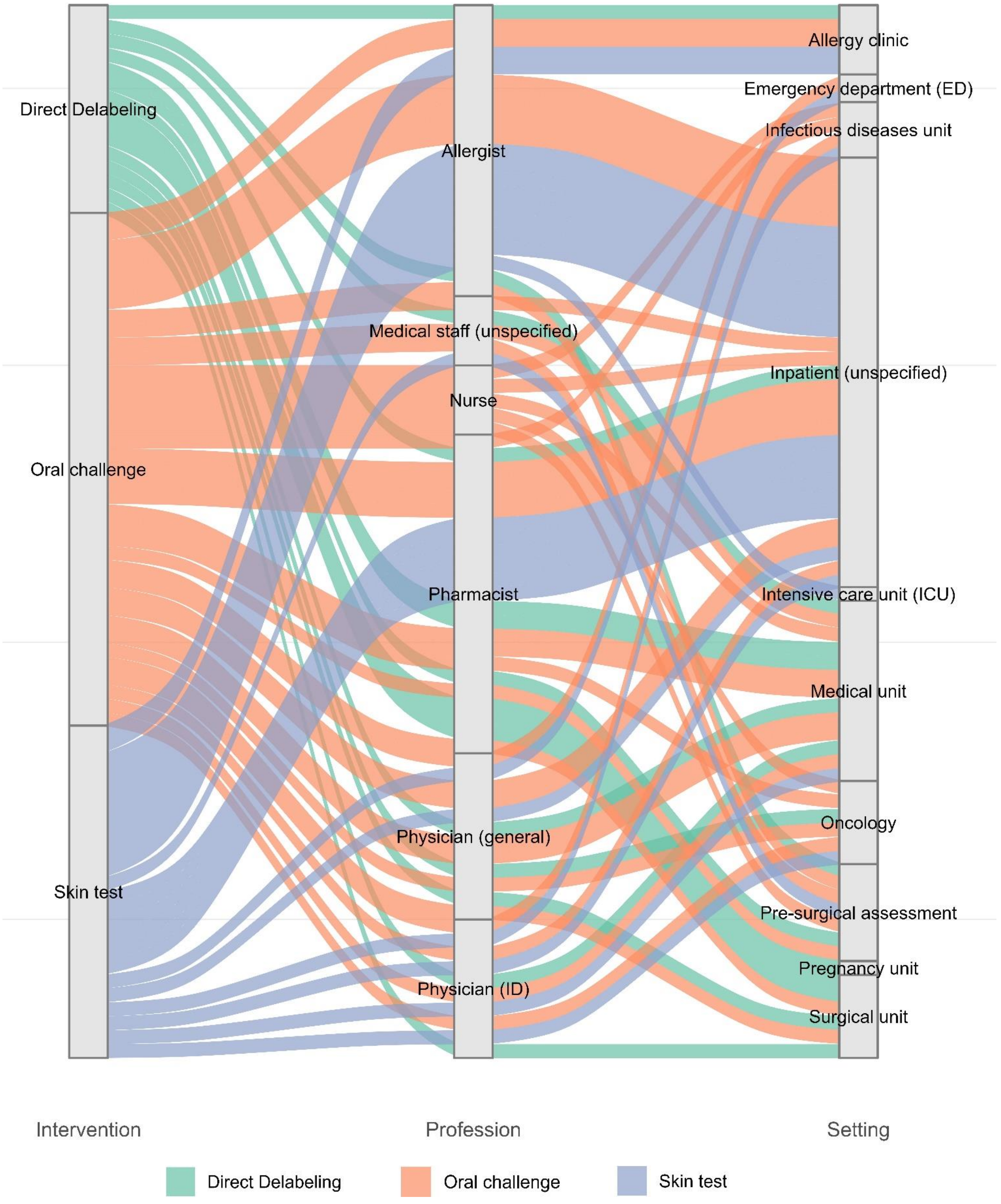

Figure S1: Interventions across healthcare providers and settings

Table S1: Search strategy

PubMed/Medline (searched on 03 September 2024)	
#1	(“Penicillins/adverse effects”[Mesh] OR “Penicillins/drug effects”[Mesh]) OR (“beta-Lactams/adverse effects”[Mesh] OR “beta-Lactams/drug effects”[Mesh])
#2	(“penicillin”[tw] OR “beta-lactam”[tw] OR “beta lactam”[tw]) AND (“allerg*”[tw] OR “reaction”[tw])
#3	(“Drug Hypersensitivity/diagnosis”[Mesh] OR “Drug Hypersensitivity/drug therapy”[Mesh] OR “Drug Hypersensitivity/prevention and control”[Mesh] OR “Drug Hypersensitivity/therapy”[Mesh] OR “Drug Hypersensitivity/organization and administration”[Mesh]) OR “Delabeling”[tw] OR “Delabelling”[tw] OR “oral challenge*”[tw] OR “Allergy test*”[tw] OR “management”[tw] OR “intervention*”[tw] OR “penicillin testing”[tw] OR “penicillin test*”[tw] OR “provocation test*”[tw] OR “provocation”[tw] OR “amoxicillin challenge”[tw] OR “penicillin challenge”[tw]
#4	#1 OR #2
#5	#3 AND #4<br>Publication Date: 1992-2024
Cochrane Library (searched on 03 September 2024)	
#1	“Penicillins” [MeSH] OR “beta lactam” [MeSH]
#2	“Drug Hypersensitivity” [MeSH]
#3	#1 AND #2
#4	Search terms: (Pencillin OR beta lactam OR beta-lactam) AND (allerg* OR reaction*)
#5	#3 OR #4
#6	Search terms: Delabel*ing OR penicillin challenge OR amoxicillin challenge OR penicillin test* OR intervention* OR management OR provocation test* OR provocation OR oral challenge OR label*ing
#7	#5 AND #6<br>Publication Date: 1992-2024
EMBASE (searched on 03 September 2024)	
#1	‘beta lactam’/exp OR ‘beta lactam’ OR ‘penicillin’ OR ‘penicillin derivative’
#2	‘allergy’ OR ‘penicillin allergy’ OR ‘drug hypersensitivity’ OR ‘allergic reaction’
#3	#1 AND #2
#4	‘delabeling’ OR ‘delabel*ing’ OR ‘amoxicillin challenge’ OR ‘penicillin challenge’ OR ‘penicillin test* OR ‘intervention’ OR ‘medication therapy management’ OR ‘provocation test’ OR ‘provocation test*’ or ‘oral challenge’
#5	#3 AND #4<br>Publication Date 1992-2024

Table S2: Detailed results of bias assessment according to GA Wells, B.S., D O'Connell, J Peterson, V Welch, M Losos, P Tugwell. The Newcastle-Ottawa Scale (NOS) for assessing the quality of nonrandomised studies in meta-analyses.

	Selection													Comparability		Outcome										Points (0-9)
Study	Representativeness of exposed cohort				Representativeness of exposed cohort			Ascertainment of exposure				Demonstration that outcome of intererst was not present at start of study		Comparability of Cohorts on the basis of design or analysis		Assessment of outcome				Was follow-up long enough for outcome to occur		Adequacy of follow up of cohorts				
	a	b	c	d	a	b	c	a	b	c	d	a	b	a	b	a	b	c	d	a	b	a	b	c	d	
Chua, K.Y.L., et al., The Penicillin Allergy Delabeling Program: A Multicenter Whole-of-Hospital Health Services Intervention and Comparative Effectiveness Study. Clin Infect Dis, 2021. 73(3): p. 487-496.	×	×	✓	×	✓	×	×	✓	×	×	×	✓	×	×	×	✓	×	×	×	✓	×	✓	×	×	×	6
Gaudreau, S., et al., Resources Assessment for Penicillin Allergy Testing Performed by Pharmacists at the Patient's Bedside. Ann Pharmacother, 2021. 55(11): p. 1355-1362.	×	✓	×	×	✓	×	×	✓	×	×	×	✓	×	×	×	×	✓	×	×	✓	×	×	✓	×	×	7
Livirya, S., et al., Oral amoxicillin challenge for low-risk penicillin allergic patients. Intern Med J, 2022. 52(2): p. 295-300.	×	×	✓	×	✓	×	×	×	✓	×	×	×	✓	×	×	✓	×	×	×	✓	×	✓	×	×	×	5
Torney, N.P. and M.D. Tiberg, Description of a pharmacist-managed/administered penicillin allergy skin testing service at a community hospital. Am J Health Syst Pharm, 2021. 78(12): p. 1066-1073.	×	✓	×	×	✓	×	×	×	✓	×	×	×	✓	×	×	✓	×	×	×	✓	×	✓	×	×	×	6
Steenvoorden, L., et al., De-labelling penicillin allergy in acutely hospitalized patients: a pilot study. BMC Infect Dis, 2021. 21(1): p. 1083.	×	×	✓	×	✓	×	×	×	✓	✓	×	×	✓	×	×	✓	×	×	✓	✓	×	✓	×	×	×	5
Savic, L., et al., Penicillin allergy de-labelling ahead of elective surgery: feasibility and barriers. Br J Anaesth, 2019. 123(1): p. e110-e116.	×	×	✓	×	✓	×	×	×	✓	×	×	×	✓	×	×	✓	×	×	✓	✓	×	✓	×	×	×	5
Moussa, Y., et al., De-labeling of beta-lactam allergy reduces intraoperative time and optimizes choice in antibiotic prophylaxis. Surgery, 2018.	✓	×	×	×	✓	×	×	✓	×	×	×	✓	×	×	×	✓	×	×	×	✓	×	✓	×	×	×	7
Song, Y.C., et al., Effectiveness and Feasibility of Pharmacist-Driven Penicillin Allergy De-Labeling Pilot Program without Skin Testing or Oral Challenges. Pharmacy (Basel), 2021. 9(3).	×	✓	×	×	✓	×	×	✓	×	×	×	×	✓	×	×	×	✓	×	×	✓	×	✓	×	×	×	6
Mann, K.L., J.Y. Wu, and S.S. Shah, Implementation of a Pharmacist-Driven Detailed Penicillin Allergy Interview. Ann Pharmacother, 2020. 54(4): p. 364-370.	×	✓	×	×	✓	×	×	✓	×	×	×	✓	×	×	×	×	✓	×	×	×	✓	✓	×	×	×	6
Ramsey, A., et al., Direct Challenges to Penicillin-Based Antibiotics in the Inpatient Setting. J Allergy Clin Immunol Pract, 2020. 8(7): p. 2294-2301.	×	✓	×	×	✓	×	×	✓	×	×	×	×	✓	×	×	✓	×	×	×	×	✓	✓	×	×	×	5
Taremi, M., et al., Safety, Efficacy, and Clinical Impact of Penicillin Skin Testing in Immunocompromised Cancer Patients. J Allergy Clin Immunol Pract, 2019. 7(7): p. 2185-2191 e1.	✓	×	×	×	✓	×	×	✓	×	×	×	×	✓	×	×	✓	×	×	×	✓	×	✓	×	×	×	6
du Plessis, T., et al., Implementation of a pharmacist-led penicillin allergy de-labelling service in a public hospital. J Antimicrob Chemother, 2019. 74(5): p. 1438-1446.	✓	×	×	×	✓	×	×	✓	×	✓	×	×	✓	×	×	×	✓	×	×	✓	×	✓	×	×	✓	6
Devchand, M., et al., Evaluation of a pharmacist-led penicillin allergy de-labelling ward round: a novel antimicrobial stewardship intervention. J Antimicrob Chemother, 2019. 74(6): p. 1725-1730.	×	✓	×	×	✓	×	×	✓	×		×	✓	×	×	×	×	✓	×	×	×	✓	×	✓	×	×	6
Ramsey, A. and M.L. Staicu, Use of a Penicillin Allergy Screening Algorithm and Penicillin Skin Testing for Transitioning Hospitalized Patients to First-Line Antibiotic Therapy. J Allergy Clin Immunol Pract, 2018. 6(4): p. 1349-1355.	×	✓	×	×	✓	×	×	×	✓	×	×	✓	×	×	×	✓	×	×	×	✓	×	✓	×	×	×	7
Marwood, J., et al., De-labelling self-reported penicillin allergy within the emergency department through the use of skin tests and oral drug provocation testing. Emerg Med Australas, 2017. 29(5): p. 509-515.	✓	×	×	×	✓	×	×	×	×	✓	×	×	✓	×	×	✓	×	×	×	✓	×	×	✓	×	×	5
Warrington, R.J., K.R. Lee, and S. McPhillips, The value of skin testing for penicillin allergy in an inpatient population: analysis of the subsequent patient management. Allergy Asthma Proc, 2000. 21(5): p. 297-9.	×	✓	×	×	✓	×	×	×	×	✓	×	×	✓	×	×	✓	×	×	×	✓	×	✓	×	×	×	5
Arroliga, M.E., et al., A prospective observational study of the effect of penicillin skin testing on antibiotic use in the intensive care unit. Infect Control Hosp Epidemiol, 2003. 24(5): p. 347-50.	✓	×	×	✓	✓	×	×	✓	×	×	×	×	✓	×	×	✓	×	×	×	✓	×	✓	×	×	×	6
DesBiens, M.T., M.S. Calderwood, and E.L. Reigh, Expanding Penicillin Allergy Evaluation in Hospitalized Patients. Am J Med, 2022. 135(8): p. 958-963 e13.	×	✓	×	×	✓	×	×	×	×	✓	×	×	✓	×	×	✓	×	×	×	✓	×	✓	×	×	×	5
Leis, J.A., et al., Point-of-Care beta-Lactam Allergy Skin Testing by Antimicrobial Stewardship Programs: A Pragmatic Multicenter Prospective Evaluation. Clin Infect Dis, 2017. 65(7): p. 1059-1065.	✓	×	×	×	✓	×	×	×	✓	×	×	×	✓	×	×	✓	×	×	×	✓	×	✓	×	×	×	6
Foolad, F., et al., The Impact of Penicillin Skin Testing on Aztreonam Stewardship and Cost Savings in Immunocompromised Cancer Patients. Open Forum Infect Dis, 2019. 6(10): p. ofz371.	×	×	✓	×	✓	×	×	×	✓	×	×	×	✓	×	×	✓	×	×	×	✓	×	✓	×	×	×	5

Trubiano, J.A., et al., Development and Validation of a Penicillin Allergy Clinical Decision Rule. JAMA Intern Med, 2020. 180(5): p. 745-752.	×	✓	×	×	✓	×	×	×	✓	×	×	×	✓	×	×	✓	×	×	×	✓	×	✓	×	×	×	6
Ham, Y., et al., Safety and efficacy of direct two-step penicillin challenges with an inpatient pharmacist-driven allergy evaluation. Allergy Asthma Proc, 2021. 42(2): p. 153-159.	×	✓	×	×	✓	×	×	×	✓	×	×	✓	×	×	×	×	✓	×	×	✓	×	✓	×	×	×	7
Harmon, S., et al., The Clinical and Financial Impact of a Pharmacist-Driven Penicillin Skin Testing Program on Antimicrobial Stewardship Practices. Hosp Pharm, 2020. 55(1): p. 58-63.	×	✓	×	×	✓	×	×	✓	×	×	×	×	✓	×	×	✓	×	×	×	✓	×	✓	×	×	×	6
Chen, J.R., et al., Improving Aztreonam Stewardship and Cost Through a Penicillin Allergy Testing Clinical Guideline. Open Forum Infect Dis, 2018. 5(6): p. ofy106.	×	✓	×	×	✓	×	×	×	✓	×	×	×	✓	×	×	×	✓	×	×	✓	×	✓	×	×	×	6
Kwiatkowski, S., et al., Optimizing preoperative antibiotics in patients with beta-lactam allergies: A role for pharmacy. Am J Health Syst Pharm, 2021. 78(Supplement_3): p. S76-S82.	✓	×	×	×	✓	×	×	✓	×	×	×	✓	×	×	×	×	✓	×	×	✓	×	✓	×	×	×	7
Brayson, J., et al., CATALYST: challenging antibiotic allergy status. J Antimicrob Chemother, 2023. 78(5): p. 1241-1244.	×	✓	×	×	✓	×	×	×	✓	×	×	×	✓	×	×	✓	×	×	×	✓	×	✓	×	×	×	6
Li, T.S., et al., Prospective Assessment of Penicillin Allergy (PAPA): Evaluating the performance of penicillin allergy testing and post-delabelling outcomes among Hong Kong Chinese. Asian Pac J Allergy Immunol, 2023.	✓	×	×	×	✓	×	×	✓	×	×	×	×	✓	×	×	✓	×	×	×	✓	×	✓	×	×	×	6
Bodega-Azuara, J., et al., Beta-lactam allergy in patients: an antibiotic stewardship challenge. Eur J Hosp Pharm, 2024. 31(4): p. 307-313.	×	✓	×	×	✓	×	×	✓	×	×	×	✓	×	×	×	✓	×	×	×	✓	×	×	✓	×	×	7
Bediako, H., et al., Impact of an inpatient nurse-initiated penicillin allergy delabeling questionnaire. Antimicrob Steward Healthc Epidemiol, 2022. 2(1): p. e86.	×	×	✓	×	✓	×	×	×	✓	×	×	×	✓	×	×	×	✓	×	×	✓	×	×	✓	×	×	5
Wade, S. and E. Marshall, A pharmacist-led penicillin allergy de-labelling project within a preoperative assessment clinic: the low-hanging fruit is within reach. J Hosp Infect, 2023. 139: p. 1-5.	×	×	✓	×	✓	×	×	✓	×	×	×	×	✓	×	×	✓	×	×	×	✓	×	×	✓	×	×	5
Copaescu, A.M., et al., Efficacy of a Clinical Decision Rule to Enable Direct Oral Challenge in Patients With Low-Risk Penicillin Allergy: The PALACE Randomized Clinical Trial. JAMA Intern Med, 2023. 183(9): p. 944-952.	×	×	✓	×	✓	×	×	✓	×	×	✓	×	✓	×	×	✓	×	×	×	✓	×	✓	×	×	×	5
Alnaes, M.B., et al., A new pathway for penicillin delabeling in Norway. World Allergy Organ J, 2023. 16(11): p. 100829.	×	✓	×	×	✓	×	×	×	✓	×	×	✓	×	×	×	✓	×	×	×	×	✓	✓	×	×	×	6
Hitchcock, A.M., et al., Impact of a Pharmacist-Conducted Preoperative Beta-Lactam Allergy Assessment on Perioperative Cefazolin Prescribing. J Pharm Pract, 2024. 37(5): p. 1073-1081.	×	✓	×	×	✓	×	×	✓	×	×	×	✓	×	×	×	×	✓	×	×	✓	×	✓	×	×	×	7
Merz, L.E., et al., Development of a Pipeline for Removing Allergy Labels in Patients Undergoing Hematopoietic Stem Cell Transplantation. Transplant Cell Ther, 2024. 30(3): p. 322 e1-322 e10.	×	✓	×	×	✓	×	×	✓	×	×	×	×	✓	×	×	×	×	✓	×	✓	×	✓	×	×	×	5
Lanoue, D., et al., Resource utilization and cost assessment of a proactive penicillin allergy de-labeling program for low-risk inpatients. Allergy Asthma Clin Immunol, 2024. 20(1): p. 7.	×	×	✓	×	✓	×	×	×	✓	×	✓	×	✓	×	×	×	✓	×	×	✓	×	✓	×	×	×	5
Krishna, M.T., et al., A multicentre observational study to investigate feasibility of a direct oral penicillin challenge in de-labelling 'low risk' patients with penicillin allergy by non-allergy healthcare professionals (SPACE study): Implications for healthcare systems. J Infect, 2024. 88(3): p. 106116.	×	×	✓	×	✓	×	×	✓	×	×	×	✓	×	×	×	✓	×	×	×	✓	×	✓	×	×	×	6
Wong, J.C.Y., et al., Prospective, Multicenter, Head-to-Head Comparison Between Allergists Versus Nonallergists in Low-Risk Penicillin Allergy Delabeling: Effectiveness, Safety, and Quality of Life (HK-DADI2). J Allergy Clin Immunol Pract, 2024. 12(7): p. 1801-1808 e2.	×	×	✓	×	✓	×	×	×	✓	×	×	×	✓	✓	×	✓	×	×	×	✓	×	✓	×	×	×	6
Sobrino-Garcia, M., et al., Delabeling of allergy to beta-lactam antibiotics in hospitalized patients: a prospective study evaluating cost savings. Int J Clin Pharm, 2024. 46(5): p. 1067-1075.	✓	×	×	×	✓	×	×	✓	×	×	×	×	✓	×	×	✓	×	×	×	×	✓	✓	×	×	×	5
Rose, M.T., et al., Oral challenge vs routine care to assess low-risk penicillin allergy in critically ill hospital patients (ORACLE): a pilot safety and feasibility randomised controlled trial. Intensive Care Med, 2024. 50(6): p. 913-921.	×	×	✓	×	✓	×	×	✓	×	×	×	✓	✓	✓	×	✓	×	×	×	✓	×	✓	×	×	×	7
Arasaratnam, R.J., et al., Rising to the Challenge: An ID Provider-Led Initiative to Address Penicillin Allergy Labels at a Large Veterans Affairs Medical Center. Open Forum Infect Dis, 2024. 11(8): p. ofae396.	×	✓	×	×	✓	×	×	✓	×	×	×	×	✓	×	×	×	✓	×	×	✓	×	✓	×	×	×	6
Drummond, K., et al., Effectiveness of direct delabelling of allergy labels in type A adverse drug reactions to penicillin: a multicentre hospitalwide prospective cohort study. J Antimicrob Chemother, 2024. 79(10): p. 2640-2644.	×	×	✓	×	✓	×	×	✓	×	×	×	✓	×	×	×	×	×	✓	×	×	✓	✓	×	×	×	4
Molina-Molina, G.J., et al., *Delabelling beta-lactam allergy.* Front Pharmacol, 2024. **15**: p. 1423719.	✓	×	×	×	✓	×	×	✓	×	×	×	✓	×	×	×	×	✓	×	×	✓	×	✓	×	×	×	7

1. Khan, D.A., et al., *Drug allergy: A 2022 practice parameter update.* J Allergy Clin Immunol, 2022. **150**(6): p. 1333-1393.
